# Clinical features of abdominal migraine: a systematic review and summary of data from 662 patients

**DOI:** 10.3389/fneur.2026.1760307

**Published:** 2026-01-29

**Authors:** Ke Ding, Hanlu Xiang, Mengjie Huo, Ke Xu, Hebo Wang

**Affiliations:** 1 Graduate School of Hebei Medical University, Shijiazhuang, Hebei, China; 2 Department of Neurology, Hebei General Hospital, Shijiazhuang, Hebei, China; 3 Graduate School of North China University of Science and Technology, Tangshan, China; 4 Hebei Provincial Key Laboratory of Cerebral Networks and Cognitive Disorders, Shijiazhuang, Hebei, China

**Keywords:** abdominal migraine, clinical features, diagnosis, migraine, systematic review, treatment

## Abstract

**Background:**

Abdominal migraine (AM) is an episodic syndrome characterized by recurrent, self-limiting episodes of abdominal pain with autonomic features, now recognized to affect both children and adults according to ICHD-3 criteria. Its diagnosis is clinical and requires the exclusion of organic gastrointestinal or renal diseases, yet no standardized treatment exists, leading to therapeutic approaches often adapted from migraine management. Challenges in diagnosis, due to difficulties in symptom description by children and cognitive biases in adults, frequently result in underdiagnosis, repeated consultations, and diminished quality of life. This study aims to analyze the clinical characteristics, diagnostic and therapeutic approaches, and outcomes of AM in pediatric and adult patients based on a large case series.

**Methods:**

A systematic literature review was conducted per PRISMA guidelines. Major databases were searched from inception to June 2025 for case reports and clinical studies on AM. Data on demographics, clinical presentation, treatment, and outcomes were extracted and analyzed.

**Results:**

We included 662 patients (629 children, 33 adults) from 63 studies. The female-to-male ratio was 1.6:1. The median age at onset was 4.2 years in children and 31.0 years in adults, with diagnostic delays of 3.1 and 4.0 years, respectively. Among cases with specific data, periumbilical pain was reported in 43.3% (of 223), while nausea (66.1%), vomiting (53.6%), and headache (47.1%) were common in a cohort of 448 cases. Photophobia, pallor, and anorexia were also frequently observed. Triptans showed the highest acute efficacy (98.04%, 50/51), versus 62.5% (5/8) for NSAIDs. Prophylactics were highly effective: anticonvulsants (95.0%, 19/20), beta-blockers (100%, 12/12), and antihistamines (92.8%, 64/69). These exceptional rates likely reflect reporting bias and require prospective validation.

**Conclusion:**

AM presents with significant clinical heterogeneity but shares core features with migraine disorders. Early diagnosis and management, potentially incorporating agents used in migraine (such as triptans and prophylactics) based on preliminary evidence, may improve outcomes, though this requires confirmation in controlled studies. Increased awareness of non-gastrointestinal symptoms and migraine history is essential for accurate diagnosis.

## 1 Background

Abdominal migraine (AM) is an episodic syndrome characterized by recurrent episodes of moderate to severe abdominal pain. These episodes are frequently associated with autonomic symptoms, including nausea, vomiting, anorexia, pallor, and photophobia. A hallmark of the condition is that each attack typically lasts between 2 to 72 h and is followed by a complete resolution of symptoms during the interictal period. Furthermore, the diagnosis necessitates the absence of evidence for organic gastrointestinal or renal pathologies based on a thorough history and physical examination (1, 2). Epidemiological studies have shown that AM predominantly affects the pediatric population, with a slight female predominance and a prevalence rate of approximately 1 to 4% (3). Notably, a recent study has reported a prevalence as high as 8.3% among children and adolescents (4). Although AM has traditionally been regarded as a pediatric disorder, there is growing recognition of its occurrence in adults. A notable proportion of patients either continue to experience episodes into adulthood or receive their initial diagnosis during adult years. The historical description of this disorder dates back to 1922, when Brams first documented the clinical manifestations of AM in adult patients (5). Over time, its classification has undergone significant evolution: in the first and second editions of the International Classification of Headache Disorders (ICHD) (6, 7), AM was categorized as a “childhood periodic syndrome,” emphasizing its association with migraine while restricting it to the pediatric population. It was not until ICHD-3 that the age restriction was removed, formally defining it as an “episodic syndrome that may be associated with migraine,” thereby expanding the diagnostic scope and acknowledging the existence of adult patients (1). The diagnosis of abdominal migraine (AM) currently relies primarily on clinical criteria, including paroxysmal abdominal pain, associated autonomic symptoms, and complete normalization between episodes. Furthermore, it is necessary to exclude other gastrointestinal or systemic organic diseases that could account for the abdominal pain. Particular attention should be paid to differentiating AM from other common periodic syndromes (e.g., cyclic vomiting syndrome and benign paroxysmal vertigo), as well as from other abdominal pain-predominant functional gastrointestinal disorders such as irritable bowel syndrome and functional dyspepsia. In terms of therapeutic management, there is currently no universally established standardized regimen for AM, and clinical practice largely draws upon treatment strategies adapted from migraine therapy (8). For acute attacks, nonsteroidal anti-inflammatory drugs (NSAIDs) or triptans may be considered. Prophylactic treatment options include beta-blockers, tricyclic antidepressants, or antiepileptic drugs such as topiramate. Non-pharmacological interventions, including lifestyle modifications, identification and avoidance of triggers, and cognitive behavioral therapy, have also shown beneficial effects in some patients. The diagnosis and management of AM continue to face significant challenges: pediatric patients are frequently misdiagnosed with other gastrointestinal disorders due to their limited ability to describe symptoms and low disease awareness, while adult patients often experience prolonged underdiagnosis or misdiagnosis owing to cognitive biases and inconsistent diagnostic criteria. These issues lead to repeated medical consultations, reduced quality of life, and even unnecessary surgical interventions. Therefore, this study aims to systematically analyze the demographic characteristics, clinical manifestations, management strategies, and prognostic outcomes in a cohort of 662 a.m. patients, including both children and adults, with the goal of systematically characterizing disease patterns in the pediatric population and providing a preliminary descriptive analysis of adult cases to inform clinical recognition and future research.

## 2 Materials and methods

### 2.1 Literature search

This systematic review was designed and performed in accordance with the Preferred Reporting Items for Systematic Reviews and Meta-Analyses (PRISMA) guidelines (9). A comprehensive literature search was conducted using the following electronic databases: PubMed, Web of Science, Embase, the Cochrane Library, Wanfang Data, the VIP Database, CBM, and the China National Knowledge Infrastructure (CNKI). Additionally, we manually searched the reference lists of relevant reviews to identify any potentially eligible studies that might have been missed by the electronic database search.

### 2.2 Methods

**Figure 1:**
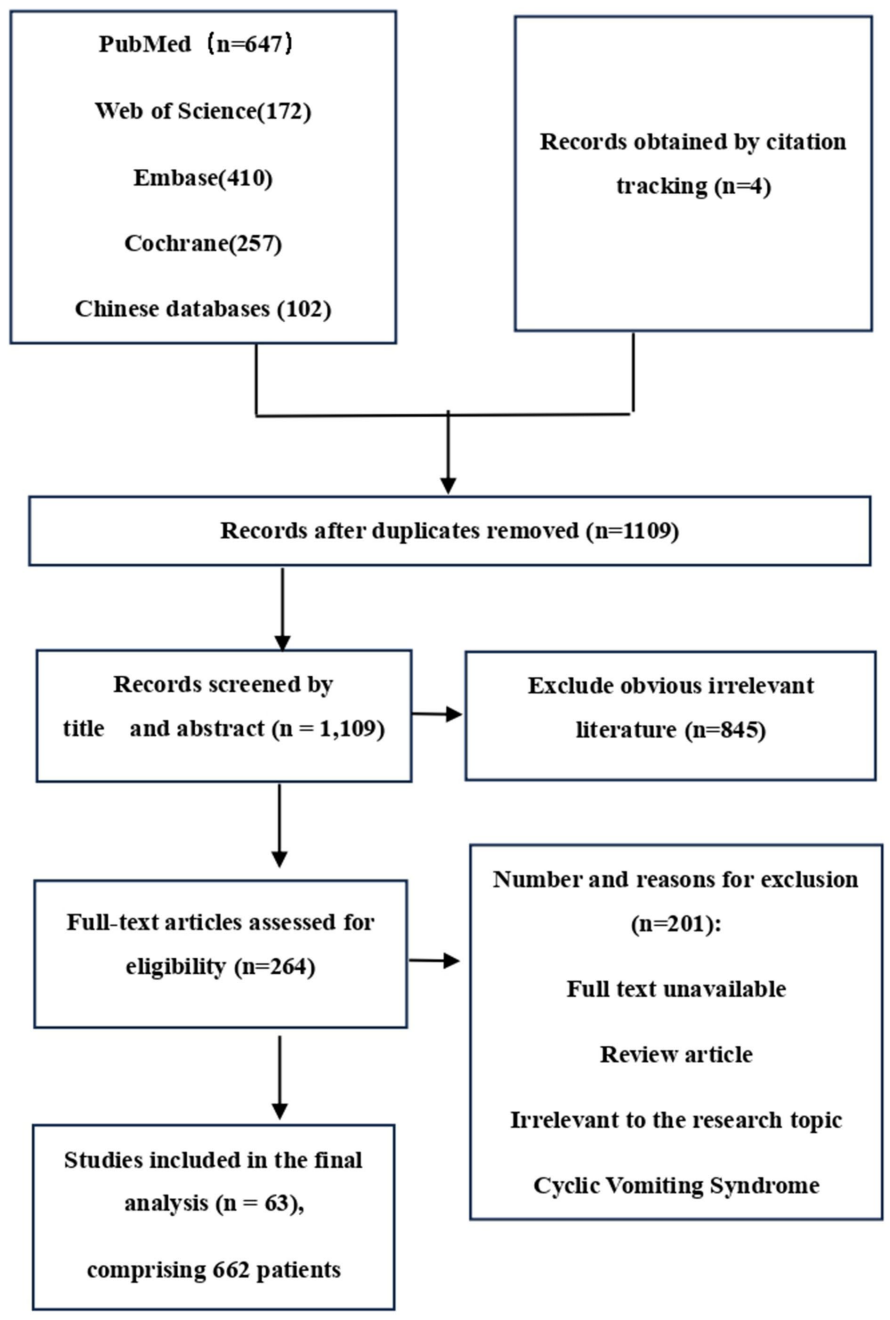
Flowchart of literature identification, screening, and inclusion.

The search term “abdominal migraine” was used to systematically retrieve case reports, reviews, and clinical studies related to AM published from database inception to June 30, 2025. The study population comprised patients with a confirmed diagnosis of abdominal migraine. The primary outcome measures included clinical features, core treatment strategies, and their effectiveness. Two authors (Ke Ding and Hanlu Xiang) independently screened the records by reviewing titles, abstracts, and full-text articles. Any disagreements regarding eligibility were resolved through discussion, with arbitration by Hebo Wang when necessary. The final included literature was determined, and the following data were extracted: author, year of publication, number of cases, age, sex, clinical characteristics of abdominal pain, associated symptoms, diagnostic and therapeutic measures, comorbidities, and disease outcomes. Studies that were irrelevant or duplicates were excluded. The initial search yielded 1,592 records of various publication types. After removing duplicates, 1,109 articles remained. Following initial and full-text screening, 63 articles (case reports and/or systematic reviews) met the inclusion criteria. Figure 1 illustrates the flowchart of literature identification, screening, and inclusion. The final study cohort comprised 662 patients derived from these 63 articles, as detailed in Table 1 (10–71). Demographic and clinical characteristics of these cases were statistically described. The methodological quality of the included case reports was assessed using the framework proposed by Murad et al. (72).

**Table 1:** The characteristics of the included studies.

First author	Year	Study title	Study design	Participants, n
Lundberg, P O	1975	Abdominal migraine--diagnosis and therapy	Case series	1
Pryszmont M	1998	Abdominal migraine in adults	Case Report	1
Onofrio FD	2006	Adult abdominal migraine: a new syndrome or sporadic feature of migraine headache? A case report	Case series	2
Newman LC	2008	Rebound abdominal pain: noncephalic pain in abdominal migraine is exacerbated by medication overuse	Case Report	1
Hamed SA	2010	A migraine variant with abdominal colic and Alice in wonderland syndrome: a case report and review	Review	1
Roberts JE	2012	Abdominal migraine, another cause of abdominal pain in adults	Case series	2
Dees B	2013	Managing migraine and other headache syndromes in those over 50	Review	1
Evans RW	2013	Cyclic vomiting syndrome and abdominal migraine in adults and children	Review	4
Woodruff AE	2013	Abdominal migraine in adults: a review of pharmacotherapeutic options	Review	1
Rasmussen E	2014	Attacks of abdominal pain can be abdominal migraine	Case Report	1
Kakisaka Y	2014	Temporal intermittent rhythmic delta activity and abdominal migraine	Case Report	1
Cervellin G	2015	Abdominal migraine in the differential diagnosis of acute abdominal pain	Case Report	1
Kunishi Y	2016	Abdominal migraine in a middle-aged woman	Case Report	1
Karimi N	2016	Adult abdominal pain; a rare cause of migraine disorders: case report	Case Report	1
Tamagawa T	2016	A case report of adult abdominal migraine	Case Report	1
Bhavesh R	2019	Adult abdominal migraine presenting with abdominal myofascial pain syndrome: case report	Case Report	1
Peng FB	2019	Chronic periumbilical abdominal pain: abdominal migraine in an adult	Case Report	1
Yuridullah R	2019	The ever-mysterious cyclical vomiting syndrome (CVS) and abdominal migraine: recognition and successful management strategies illustrated by a case report	Case Report	1
Karmali R	2020	Fifty-eight-year-old female with abdominal migraine: a rare cause of episodic gastrointestinal disturbance in adults	Case Report	1
Hermanowicz N	2021	Adult abdominal migraine improved by onabotulinumtoxinA injections	Case Report	1
von Muhlenbrock C	2022	Abdominal migraine, unusual cause of chronic abdominal pain in adults	Case Report and Review	1
Diao S	2022	An adult case of abdominal migraine manifested mainly as cyclic abdominal pain with nausea and vomiting	Case Report	1
Zhao W	2023	Adult abdominal migraine: a case report and literature review	Case Report and Review	1
Wang C	2021	Adult abdominal migraine: a case report and literature review	Case Report and Review	1
Sakai F	2025	The Potential Link Between Abdominal Migraine and Chronic Apical Periodontitis: A Case Repor	Case Report	1
Hazzouri R	2024	Unveiling the Enigma: Abdominal Migraine in Adults - A Case Report and Discussion	Case Report	1
Baskaran T	2025	Calcitonin gene-related peptide-targeting drugs efficiently treat abdominal migraine—A case report	Case Report	1
Lundberg P O	2010	Abdominal migraine: diagnosis and therapy	Case Report	1
Worawattanakul M	1999	Abdominal Migraine: Prophylactic Treatment and Follow-up	Case Report	1
Ibrahim M	2023	Abdominal Migraines: A Rare Adulthood Manifestation of a Typical Childhood Disease	Case Report	1
Monteferrante NR	2021	Prevention of Perioperative Abdominal Migraine in a Patient Undergoing Spinal Fusion: A Case Report	Case Report	1
Wu Y	2023	HU Siyuan’s experience in treating pediatric abdominal migraine from the perspective of liver and spleen	Experience Summary	1
Ye C	1994	Clinical epidemiology of pediatric abdominal migraine in a general urban clinic	Epidemiological Study	26
Tan V	2006	Abdominal migraine and treatment with intravenous valproic Acid	Case Report	2
Piper D W	1951	Abdominal migraine; report of a case	Case Report	1
Kakisaka Y	2010	Abdominal migraine associated with ecchymosis of the legs and buttocks: does the symptom imply an unknown mechanism of migraine?	Case Report	1
Heuschkel R	2001	Abdominal Migraine in Children With Neurofibromatosis Type 1: A Case Series and Review of Gastrointestinal Involvement in NF1	Case Series	6
Devanarayana NM	2016	Abdominal migraine in children: association between gastric motility parameters and clinical characteristics	Observational Study	17
Catala-Beauchamp A	2012	Abdominal Migraine in Children: Is It All in Their Heads?	Review	1
Amin MA	2024	Abdominal migraine with acute watery diarrhea and dehydration: Successful treatment with Valproic acid in a pediatric case	Case Report	1
Carson L	2011	Abdominal migraine: an under-diagnosed cause of recurrent abdominal pain in children	Retrospective Cohort Study	20
Hawa K	2020	Abdominal migraines: Variations in diagnosis and care between pediatric gastroenterologists and neurologists	Cross-sectional Study	69
Prichard J S	1958	Abdominal pain of cerebral origin in children	Case Series	17
Devanarayana NM	2011	Abdominal pain-predominant functional gastrointestinal diseases in children and adolescents: prevalence, symptomatology, and association with emotional stress	Cross-sectional Study	21
Altamimi E M	2014	Abdominal Pain-Predominant Functional Gastrointestinal Disorders in Jordanian School Children	Cross-sectional Study	36
Gu D	2020	Case for abdominal migraine in child patient treated by acupuncture	Case Report	1
Lee K	2013	The clinical characteristics of abdominal migraine and risk factors for developing migraine later in childhood	Cohort Study	84
Mortimer MJ	1993	Clinical epidemiology of childhood abdominal migraine in an urban general practice	Epidemiological Study	26
Abu-Arafeh IA	1993	Current controversies--abdominal migraine	Opinion	20
Kakisaka Y	2009	Efficacy of sumatriptan in two pediatric cases with abdominal pain-related functional gastrointestinal disorders: does the mechanism overlap that of migraine?	Case Report	2
Rahmani P	2020	Evaluating the effects of probiotics in pediatrics with recurrent abdominal pain	Randomized Controlled Trial	9
Daga S	2024	Fluoxetine for the Treatment of Abdominal Migraine	Correspondence	1
Oswari H	2019	Functional abdominal pain disorders in adolescents in Indonesia and their association with family related stress	Cross-sectional Study	8
Barron SA	1999	Index of suspicion. Case 2. Abdominal migraines	Case Report	1
Symon DN	1992	Is there a place for “abdominal migraine” as a separate entity in the IHS classification? Yes!	Controversies	120
Teixeira K C S	2014	Migraine equivalents in childhood	Retrospective Cohort Study	15
Al-Twaijri WA	2002	Pediatric migraine equivalents: occurrence and clinical features in practice	Observational Study	20
Hikita T	2016	Prevalence of abdominal migraine and recurrent abdominal pain in a Japanese clinic	Cross-sectional Study	7
Al Lawati TT	2022	Prophylactic Therapy Response in Children with Abdominal Migraine: A Single Centre Experience in Oman	Retrospective Study	43
Popovich DM	2010	Recognizing and diagnosing abdominal migraines	Review	1
Quak S H	2015	Recurrent abdominal pain in children: a clinical approach	Observational Study	20
ÖKSÜZ N	2020	Unusual Primary Headaches of Children and Adolescents: Practical tips for physicians	Review	1
Mortimer MJ	1990	The VER as a diagnostic marker for childhood abdominal migraine	Case–control study	28

### 2.3 Statistical analysis

All statistical analyses were performed using IBM SPSS Statistics version 21.0. Continuous variables following a normal distribution were expressed as mean ± standard deviation (x̄ ± SD), while those not conforming to normality were presented as median with interquartile range (IQR). Categorical variables were summarized as frequencies and percentages [n (%)]. Owing to study heterogeneity and the lack of controlled data, a formal meta-analysis was not conducted; instead, a comparative descriptive analysis of pediatric and adult subgroups was performed.

## 3 Results

### 3.1 Demographic characteristics

**Figure 2:**
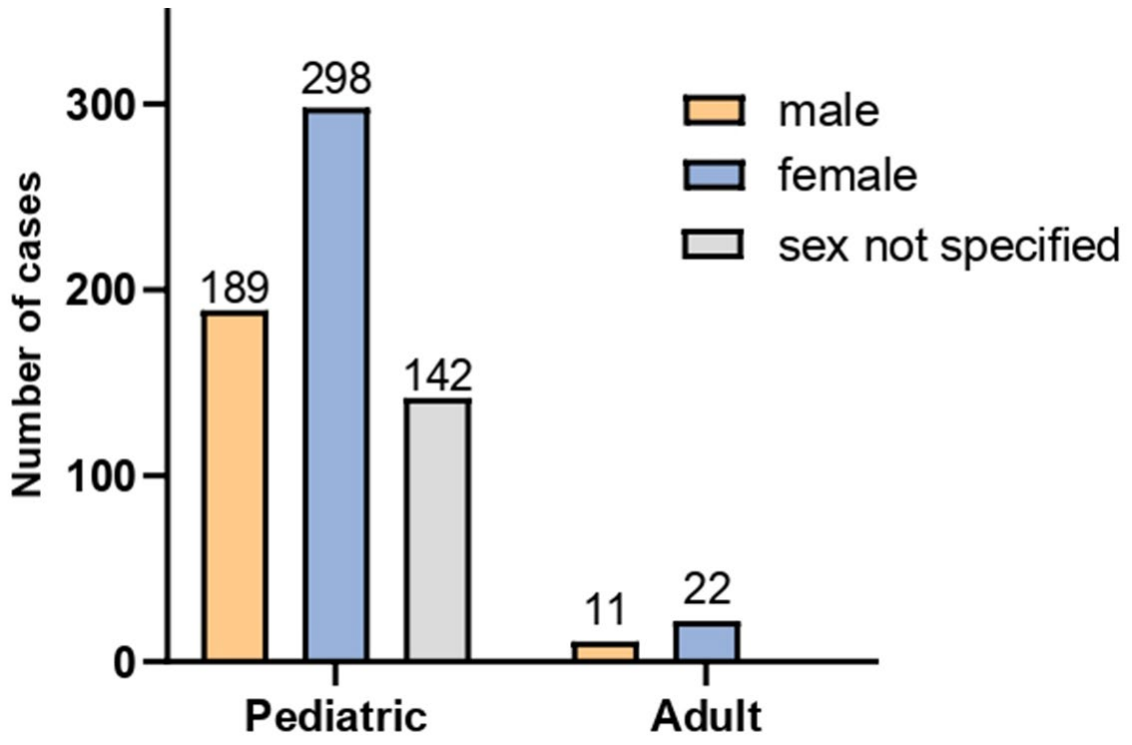
Sex distribution of the 662 patients with abdominal migraine.

A total of 662 patients with abdominal migraine were included in this study, comprising 629 patients in the pediatric group (defined as <18 years) and 33 adults. Among them, 200 (30.2%) were male and 320 (48.3%) were female, while gender information was missing for 142 patients (21.5%), all of whom were in the pediatric group. Of the 520 patients with available gender data, the overall female-to-male ratio was approximately 1.60:1. Stratified analysis revealed a female-to-male ratio of approximately 2:1 in the adult group and 1.57:1 in the pediatric group (Figure 2).

Analysis of age characteristics revealed distinct profiles between groups. In the pediatric cohort (*n* = 629, <18 years), the median age at diagnosis was 7.20 years (IQR: 7.10–9.70; range: 5–17), while the median age at onset was 4.20 years (IQR: 4.00–6.00; range: 0.5–14). The median diagnostic delay in this group was 3.10 years (IQR: 1.90–5.80), with a maximum delay of 14.5 years. In the adult group (n = 33, ≥18 years), the median age at diagnosis was 40.00 years (IQR: 27.00–52.00; range: 20–74). The median age at onset was 31.00 years (IQR: 19.00–48.00; range: 8–58), with a median diagnostic delay of 4.00 years (IQR: 2.00–8.00). Among adults, 8 patients (22.2%) were diagnosed within 1 year of onset, contrasting with a maximum delay of 58 years. Based on the age at onset, 72.2% of adult cases (*n* = 26) were classified as adult-onset (≥18 years), while 20.6% (*n* = 7) were classified as childhood-onset. The specific onset ages for the latter subgroup were 8, 9, 10, 14, 15, 15, and 16 years. In this study, 310 out of 662 patients with abdominal migraine (46.83%) had a personal and/or family history of migraine, with a predominance of maternal inheritance. Among the 33 adult patients, this proportion was significantly higher, reaching 27 cases (82%). Specifically, 17 patients (51.5%) had a family history of migraine, while 20 patients (60.6%) had a personal history of migraine. Among those with a personal migraine history, three (15%) had pediatric-onset migraine that resolved in adulthood, while the remaining 17 patients (85%) experienced symptoms that persisted into adulthood. The following descriptions of clinical features, management, and outcomes include data from the adult subgroup (*n* = 33). Given the limited sample size and the case-report nature of these data, findings specific to adults should be interpreted as descriptive and exploratory.

### 3.2 Clinical features

#### 3.2.1 Quality of abdominal pain

Among the 55 patients with documented descriptions of pain quality, the reports were heterogeneous: colicky pain was most common (40%), followed by dull pain (32.7%). Pressing sensation and stabbing pain were less frequent, each comprising 3.6% of cases. The remaining 20.1% were classified as other types, including non-specific descriptors such as poorly characterized discomfort, burning sensation, or pulsating pain.

#### 3.2.2 Location of abdominal pain

Among the 223 patients with documented descriptions of pain location, the most frequently reported pain locations were periumbilical (43.3%) and midline (23.6%). Other locations included diffuse pain (4.3%), pain in the left lower quadrant (2.6%), and non-localizable pain (1.7%). Less common sites were the left flank (*n* = 3), and the left upper quadrant, right lower quadrant, and lumbar region (one case each).

#### 3.2.3 Pain intensity

Among the 173 patients with documented pain severity, the majority (83.2%) experienced moderate to severe pain, including 2.9% with extreme severity, while 6.4% reported mild abdominal pain. Additionally, in 10.4% of cases involving young children who were unable to self-report, the pain was classified as moderate to severe based on behavioral indicators such as inhibition of normal activities and crying episodes.

#### 3.2.4 Duration and frequency of abdominal pain episodes

The median duration of abdominal pain episodes was 13.30 h (IQR: 1.60–24.00). The majority of patients (75.3%) experienced episodes lasting between 2 and 72 h, while 24.1% had episodes shorter than 2 h. Three patients (0.6%) reported prolonged episodes exceeding 72 h (up to 4–5 days). The frequency of episodes varied considerably, ranging from once every 4–6 months to more than 20 episodes per month.

#### 3.2.5 Associated symptoms

Among the 448 patients with documented information on accompanying symptoms, the most common accompanying symptoms included nausea (66.1%), vomiting (53.6%), headache (47.1%), pallor (31.9%), and loss of appetite (24.3%). Other frequently reported symptoms were limb pain (16.8%), photophobia (15.6%), phonophobia (8.3%), fatigue (6.3%), and diarrhea (3.1%). Headache was reported in 47.1% (211/448) of patients. Detailed characterization of these headaches in the source literature was limited. Where specified, the temporal relationship varied: headaches could occur concurrently with, or precede abdominal pain episodes, and did not accompany every attack in affected individuals (23, 47). Notably, two cases explicitly described migrainous headaches preceding abdominal pain (12, 30). For the majority of reports, however, the accompanying headaches were not described as migrainous in nature. The original studies did not systematically apply ICHD-3 criteria to classify headache type. Notably, limb pain and fever (7.6%) were reported exclusively in pediatric patients, with no such cases observed in adults. Less commonly observed symptoms included flushing (0.9%) and aggressive behavior (0.7%). Additionally, isolated cases accompanied by skin bruising, blurred vision, olfactory abnormalities, auditory hallucinations, transient loss of consciousness, and thirst were documented. Detailed statistical data regarding the clinical characteristics are presented in Table 2.

**Table 2:** Analysis of clinical characteristics in abdominal migraine.

Patient characteristic	Value, *n* (%)
Site of abdominal pain (*n* = 233)	
Periumbilical	101 (43.3)
Midline	55 (23.6)
Epigastric	35 (15.0)
Lower abdomen	17 (7.3)
Diffuse/Generalized	10 (4.3)
Left lower quadrant	6 (2.6)
Poorly localized	4 (1.7)
Nature of abdominal pain (*n* = 55)	
Colicky pain	22 (40.0)
Dull pain	18 (32.7)
Sense of pressure	2 (3.6)
Stabbing pain	2 (3.6)
Associated symptoms (*n* = 448)	
Nausea	296 (66.1)
Vomiting	240 (53.6)
Anorexia	109 (24.3)
Headache	211 (47.1)
Photophobia	70 (15.6)
Phonophobia	37 (8.3)
Pallor	143 (31.9)

#### 3.2.6 Aura and prodromal symptoms

A total of 26 patients experienced heterogeneous aura or prodromal symptoms prior to attacks. Adult patients exhibited diverse and atypical manifestations beyond common symptoms such as nausea, vomiting, photophobia, and phonophobia. These included paresthesia, right scapular pain, cold or burning sensations in the chest, skin flushing, and hypoesthesia of the tongue and occipital region. In contrast, symptoms in pediatric patients were more homogeneous and predominantly included mood disturbances such as depression and irritability (15 cases, 60.0%). Non-specific prodromes such as fatigue (1 case) and nausea/vomiting (1 case) were less frequently reported. Notably, 4 pediatric patients (16.0%) exhibited typical migraine-like visual or sensory aura.

#### 3.2.7 Triggers and alleviating factors

The triggers and alleviating factors for abdominal migraine are similar to those of classic migraine. Common triggers include psychological stress, sleep deprivation, fatigue, alcohol, and specific foods, isolated cases have also reported anesthetics and opioids as potential triggers. Alleviating factors consist primarily of sleep and vomiting. In addition, some adult female patients experienced symptom relief during pregnancy. An observational study of 44 pediatric abdominal migraine patients further suggested a potential temperature-related alleviating effect, showing that 50% (22/44) of children remained free of attacks during summer. A similar pattern was observed in one adult patient, in whom symptoms were triggered by cold exposure and alleviated by hot water immersion.

#### 3.2.8 Auxiliary examination

The majority of patients showed no significant abnormalities on physical examination, though some exhibited abdominal tenderness during attacks. Routine auxiliary examination, including laboratory tests, imaging studies, and select gastrointestinal endoscopies, were largely negative. A few cases reported specific findings: delayed gastric emptying was observed in 18 pediatric patients, and electroencephalography (EEG) showed increased slow-wave activity in 4 children. Of particular importance, a prospective observational study of 28 children with abdominal migraine found that 27 (96.4%) exhibited significantly altered responses to red and white flash visual evoked potentials (VEP) during the interictal period compared to healthy controls.

#### 3.2.9 Management

When evaluating the efficacy of monotherapy or combination therapy (for both acute and prophylactic treatment), we only included data from drugs used in at least five patients. Previous history of treatment failure was fully considered to avoid overestimation of efficacy. It is important to note that the efficacy data were primarily derived from uncontrolled case reports and case series. The results demonstrated an overall response rate of 98.04% (50/51 patients) for triptans. Non-steroidal anti-inflammatory drugs (NSAIDs) showed an overall response rate of 62.5% (5/8 patients). For conventional migraine prophylactic medications, anticonvulsants (notably topiramate) yielded a response rate of 95.0% (19/20 patients), beta-blockers 100% (12/12 patients), tricyclic antidepressants 88.9% (8/9 patients), calcium channel blockers 90.0% (9/10 patients), and antihistamines 92.8% (64/69 patients), with the aggregate response rate exceeding 85%. In addition, case reports suggest that calcitonin gene-related peptide (CGRP) targeted agents may have potential in AM treatment. Sporadic cases have also reported benefits from alternative therapies such as acupuncture and Chinese herbal medicine.

#### 3.2.10 Outcomes

In this cohort, abdominal migraine symptoms resolved in five pediatric patients between the ages of 10 and 18 years (specific ages: 10, 14, 16, 18, and 18). Seven patients developed migraine headaches in adulthood. Additionally, one patient developed comorbid migraine with sensory aura starting at the age of 21, with abdominal pain episodes occurring following the onset of migraine attacks.

#### 3.2.11 Comorbidities

Common comorbidities associated with abdominal migraine include anxiety and depression, motion sickness, and a history of allergies. This cohort study revealed that anxiety and depression were predominantly observed in adult patients, whereas motion sickness and allergic history were more frequently reported in pediatric cases. Additionally, a spectrum of rare comorbidities was documented, including neurofibromatosis type 1, recurrent limb pain, Alice in Wonderland syndrome, attention-deficit/hyperactivity disorder (ADHD), and Dandy-Walker syndrome.

### 3.3 Quality assessment of included studies

The methodological quality of the included case reports was assessed using the framework proposed by Murad et al., which evaluates four domains: case selection, ascertainment of exposure, causality assessment, and detail of reporting. Based on these criteria, all included studies were judged to be of good overall quality.

## 4 Discussion

AM is a periodic syndrome closely related to migraine (73), and has been incorporated into the International Classification of Headache Disorders. It is also recognized in the field of gastroenterology and is included in the Rome IV diagnostic criteria as a category of functional gastrointestinal disorders (FGIDs) in children (74). A comparison of the diagnostic criteria between ICHD-3 and Rome IV is presented in Table 3. To date, no comprehensive consensus has been established regarding its clinical characteristics and management strategies. By integrating data from 662 patients with abdominal migraine (AM), including 629 children and 33 adults, this study provides the first comprehensive characterization of both the similarities and differences in clinical features between pediatric and adult AM populations, offering a preliminary evidence-based foundation for addressing current challenges in the diagnosis and management of this condition.

**Table 3:** Comparison of ICHD-3 vs. Rome IV diagnostic criteria.

ICHD-3^a^	Rome IV^b^
At least five attacks of abdominal pain, fulfilling criteria A–C	Diagnostic criteria must include all of the following ***(Criteria must be fulfilled at least twice in the preceding 6 months)***
A. Pain has at least two of the following three characteristics: 1. midline location, periumbilical or poorly localized 2. dull or ‘just sore’ quality 3. moderate or severe intensity	A. Paroxysmal episodes of intense, acute periumbilical, midline, or diffuse abdominal pain, lasting 1 h or more (refers to the most severe and distressing symptom)
B. Attacks last 2–72 h when untreated or unsuccessfully treated	B. A stereotypical pattern and symptoms for the individual patient.
C. At least two of the following four associated symptoms or signs: 1. Anorexia 2. Nausea 3. Vomiting 4. Pallor	C. The pain is associated with 2 or more of the following: 1. Anorexia 2. Nausea 3. Vomiting 4. Headache 5. Photophobia 6. Pallor
D. Complete freedom from symptoms between attacks	D. The pain interferes with normal activities or causes disability
E. Not attributed to another disorder	E. Episodes are separated by weeks to months
	F. After appropriate evaluation, the symptoms cannot be fully explained by another medical condition

^a^ICHD-3: The International Classification of Headache Disorders-3. ^b^Rome IV: Functional Gastrointestinal Disorders Rome IV.

In this cohort, pediatric patients accounted for 95% (629/662) of the study population, indicating that abdominal migraine (AM) is far more common in children than in adults. However, since the publication of the ICHD-3 criteria in 2018, there has been a noticeable increase in reported adult AM cases, suggesting that AM in adults is gradually gaining clinical recognition. Nevertheless, significant knowledge gaps regarding AM remain. This study found that the median age of onset in pediatric patients was 4.2 years, with a diagnostic delay of 3.1 years, highlighting substantial delays in diagnosis in this population, a phenomenon that may be partly attributable to limited self-reporting abilities in young children. Although adult AM cases constituted only 5% (33/662) of the cohort, they experienced an even longer median diagnostic delay of 4.0 years, with the longest case reaching 58 years (12). Such delays may lead to misdiagnosis and unnecessary surgical interventions, causing substantial physical and psychological distress to patients and imposing a significant economic burden on both individuals and society. Therefore, improving early recognition and standardized management of AM may alleviate patient suffering and prevent unnecessary surgeries resulting from underdiagnosis.

Among patients with documented sex information, the overall female-to-male ratio was approximately 1.6:1, with similar ratios observed in both the adult (2:1) and pediatric (1.57:1) subgroups. This consistency with the female predominance in migraine suggests a potential role of estrogen-mediated mechanisms. The primary locations of abdominal pain were the periumbilical region (43.3%) and midline area (23.8%), consistent with the core descriptors in the ICHD-3 criteria. However, this study also identified non-classical pain localizations, including the upper abdomen (15.2%), lower abdomen (7.4%), and diffuse pain (3.9%), none of which are currently encompassed by existing diagnostic criteria. Similarly, pain character exhibited notable heterogeneity: although adult patients provided more precise descriptions, both children and adults most frequently reported dull pain (33.3%) and colicky pain (38.9%). Other documented qualities such as pressing sensation (3.7%) and stabbing pain (3.7%) are also not included in the standard criteria. These heterogeneous features contribute to a significantly lower alignment between clinical presentations and current diagnostic requirements regarding pain location and character. This study found that 93.6% of patients (including non-verbal children) experienced moderate to severe pain. Notably, approximately 10% of young children, though unable to self-report pain intensity, exhibited behavioral indicators, such as inhibition of daily activities and crying, objectively indicating pain of at least moderate severity. The current ICHD-3 criteria rely solely on subjective pain descriptions, which may lead to the exclusion of children incapable of articulating their pain. Based on these findings, we propose that “pain resulting in functional impairment” be incorporated as a diagnostic requirement in future revisions of the criteria. Nausea (65.9%) and vomiting (53.4%) were the most prevalent gastrointestinal accompaniments of abdominal migraine, consistent with ICHD-3 diagnostic criteria. Other gastrointestinal symptoms, such as changes in stool consistency or frequency, were less frequently observed. Notably, headache was reported in 47.1% of the cohort, a prevalence significantly higher than that of anorexia (24.4%) or pallor (32.1%) (χ^2^ = 38.7, *p* < 0.001). In patients with accompanying headache, abdominal pain and headache frequently coincided, although headache did not accompany every episode of abdominal pain. Additionally, headaches could also manifest during interictal periods of abdominal pain. Furthermore, the majority of headaches reported were non-migrainous in nature. Headaches could either precede the onset of abdominal pain or occur concurrently within the same episode. In light of these findings, some experts have proposed including headache as an associated symptom to broaden the diagnostic criteria for abdominal migraine (75). Interestingly, according to the ICHD-3 diagnostic criteria, a diagnosis of abdominal migraine requires that episodes occur “in the absence of headache” or that any associated headache “does not fulfill criteria for migraine.” This stipulation implies, by definition, that if an abdominal episode is accompanied by headache meeting migraine criteria, the event should be classified as a migraine attack rather than abdominal migraine. In such cases, the abdominal pain is more appropriately regarded as a non-headache manifestation of migraine or a “migraine equivalent.” This interpretation is supported by experts in the field, who consider abdominal migraine a precursor or variant manifestation of migraine rather than an independent disorder (76). Research on the association between migraine and recurrent abdominal pain is accumulating (10, 66, 73, 77–81). One study demonstrated that the prevalence of migraine in children with abdominal migraine was more than twice that in the general pediatric population (73). Conversely, the rate of abdominal migraine in children with migraine was also more than double that observed in the general pediatric population. A similar pattern was observed in our cohort: the proportion of patients with a personal and/or family history of migraine was as high as 46.83%, which is significantly higher than the reported prevalence of migraine in the general population (11.6%) (82). Therefore, in the clinical evaluation of patients with abdominal pain–predominant functional gastrointestinal disorders (AP-FGIDs), particular attention should be paid to non-GI accompanying symptoms as well as personal or family history of migraine. In practice, however, because most patients present with moderate to severe abdominal pain, extra-intestinal symptoms are often underreported or overlooked. Consequently, these patients frequently undergo primary evaluation in departments of Gastroenterology or Emergency Medicine rather than Neurology, which may contribute to underdiagnosis or misdiagnosis.

In this cohort, a subset of patients with abdominal migraine was observed to experience a range of prodromal symptoms, such as fatigue, lethargy, and mood changes, as well as aura-like phenomena, including paresthesia, prior to the onset of abdominal pain. Additionally, atypical manifestations potentially related to autonomic dysfunction, such as flushing, localized pain, or cold/burning sensations, were also documented. Together, these symptoms constitute a complex premonitory phase preceding the acute attack. Although both adult and pediatric patients with abdominal migraine experience prodromal symptoms, their manifestations exhibit distinct age-related patterns. Adult patients more frequently report specific somatosensory abnormalities, such as well-localized paresthesia, scapular pain, or thermal sensations (e.g., cold or burning), which may reflect their more developed cognitive and descriptive abilities (17). In contrast, pediatric prodromal presentations are often non-specific, predominantly comprising emotional and behavioral changes, noted in 60.0% of cases, including irritability, depressed mood, reduced activity, or crying (56). Typical visual or sensory aura, although reported, was relatively uncommon in children. The observed differences in prodromal symptom presentation between adults and children are more likely attributable to variations in neurodevelopmental maturity, symptom perception, and verbal expression capabilities, rather than representing distinct underlying pathophysiologies. In children, particularly those of young age, the limbic system and emotional centers of the brain are relatively well-developed, whereas the parietal cortex, which is responsible for the precise perception and description of interoceptive sensations, as well as language function remain immature. Their difficulty in articulating specific interoceptive sensations like paresthesia or thermal dysesthesia often leads them to externalize the discomfort through broader emotional and behavioral disturbances. Therefore, emotional-behavioral changes in children and somatosensory abnormalities in adults may share a common underlying pathophysiology, such as cortical spreading depression (CSD) or dysregulation of brainstem/hypothalamic regulatory mechanisms, manifesting as age-dependent clinical features.

Approximately 9% of patients in this cohort exhibited relatively identifiable triggering and alleviating factors. Common triggers included psychological stress, sleep disturbances, and specific dietary items such as chocolate, red wine, cheese, and pickled foods. Alleviating factors were sleep/rest and vomiting. This pattern closely overlaps with that of migraine (73), further supporting the concept that abdominal migraine belongs to the same disease spectrum. Notably, the phenomenon of “thermal alleviation” was particularly prominent in the pediatric population, with 50% of affected children experiencing no attacks during summer, providing preliminary evidence for environmental intervention strategies such as thermal regulation therapy. Moreover, this phenomenon was also documented in one adult male patient who reported clearly cold-triggered abdominal migraine attacks that were alleviated following warm water immersion (35). This study reports a case of an adult female patient with abdominal migraine who experienced marked symptom alleviation during pregnancy (21). Although based on a single case, this observation is consistent with the well-documented remission of classical migraine during gestation (83), a phenomenon widely attributed to profound changes in the hormonal milieu throughout pregnancy.

Although routine auxiliary examinations (e.g., imaging and endoscopy) were mostly negative, a single observational study involving 28 children with abdominal migraine reported a high interictal visual evoked potential (VEP) abnormality rate of 96.4% (71). This preliminary finding suggests that altered cortical excitability within the visual pathways could be investigated further as a potential feature of AM. It highlights VEP as a candidate tool for objective assessment in future research, particularly in pediatric patients with diagnostic challenges. Furthermore, sporadic reports of delayed gastric emptying (20, 42, 43, 51), and increased slow-wave activity on electroencephalography (42, 43, 46) suggest that gastrointestinal motility and abnormal neuroelectrical activity may be involved in the pathological process of AM. However, further large-scale studies are needed to confirm the generalizability of these findings. This study aggregates the largest case series of adult AM to date (n = 33), providing a first descriptive overview of this population. However, the sample remains small and derived solely from case reports, implying selection bias and unstable estimates. These findings should therefore be considered hypothesis-generating, not definitive. No direct statistical comparisons were made with the pediatric cohort due to fundamental sample and design disparities. Robust conclusions about adult AM require future studies in larger, prospective cohorts.

This study compiled response rate data from the available literature, which were primarily derived from case reports and case series. In the context of acute treatment, based on the data included in this review, the reported response rate for triptans (98.04% [50/51]) was numerically higher than that for NSAIDs (62.5% [5/8]). These rates must, however, be interpreted with caution. The exceptionally high response rates, particularly for triptans and preventive medications such as beta-blockers (100% [12/12]), anticonvulsants (95.0% [19/20]), and antihistamines (92.8% [64/69]), likely reflect substantial publication and selection biases inherent in the included study designs, for instance, the preferential reporting of positive outcomes, rather than established efficacy at the population level. Therefore, while these pooled results suggest potential clinical utility and align with the hypothesis that AM and migraine may share common neurological targets, they do not constitute a robust evidence base for recommending first-line treatment. Prospective controlled trials are urgently needed to verify these preliminary observations. It is noteworthy that the efficacy of CGRP-targeted agents in individual cases (34), suggests their potential as a therapeutic option for AM, although this likewise requires further validation through prospective studies. On the other hand, although sporadic reports have indicated benefits with alternative therapies, such as acupuncture and Chinese herbal medicine, the limited evidence base necessitates cautious interpretation. In our cohort, the symptoms of five pediatric AM cases resolved during late adolescence (ages 10–18), aligning with the recognized self-limiting nature of the condition with age. However, the progression to migraine in seven cases, alongside one case presenting with comorbid abdominal pain and migraine, suggests that AM may represent an early manifestation within the migraine life cycle (84). Alternatively, abdominal migraine itself could be considered a prodromal manifestation of migraine in these patients. Comorbidity analysis further revealed that anxiety and depression were significantly associated with adult AM, whereas motion sickness and a history of allergies were more prevalent in pediatric patients. This distinct, age-stratified comorbidity pattern highlights the necessity of integrating psychological assessment into the management of adults with AM, while underscoring the importance of vestibular function evaluation and allergen screening in pediatric cases. Although rare comorbidities such as Alice in Wonderland Syndrome (14) and Dandy-Walker syndrome (70) were reported only as individual cases, they nonetheless suggest a potential link between AM and the central nervous system (85).

This study, based on the largest AM cohort to date (*n* = 662), provides a comprehensive characterization of the pediatric phenotype and a preliminary description of adult presentations, covering core clinical features and management strategies. Nevertheless, it has several limitations. The relatively small sample size of the adult subgroup (*n* = 33) may limit the generalizability of the findings in this population and underscores the need for prospective studies specifically focused on adults. Some data relied on retrospective records, which are susceptible to recall bias and incomplete documentation. Additionally, as the diagnostic criteria for abdominal migraine (e.g., Rome and ICHD classifications) have been refined over the decades covered by this review, some heterogeneity in case definitions across studies is an inherent feature of the available evidence. In conclusion, the clinical evaluation of suspected AM should place particular emphasis on non-gastrointestinal symptoms, such as photophobia, phonophobia, and headache, as well as on personal and family history of migraine. Following appropriate assessment, a therapeutic trial of triptans, or even CGRP-targeted agents in select cases, may be considered. Given that early diagnosis and intervention are crucial for a favorable prognosis, increased recognition and awareness of AM among clinicians are paramount.

## Data Availability

The original contributions presented in the study are included in the article/supplementary material, further inquiries can be directed to the corresponding author.
